# Vascular Calcification and Stone Disease: A New Look towards the Mechanism

**DOI:** 10.3390/jcdd2030141

**Published:** 2015-06-25

**Authors:** Allen J. Yiu, Daniel Callaghan, Razia Sultana, Bidhan C. Bandyopadhyay

**Affiliations:** 1 Calcium Signaling Laboratory, Research Service, Veterans Affairs Medical Center, 50 Irving Street, NW, Washington, DC 20422, USA; E-Mails: allen.yiu@va.gov (A.J.Y.); danieljcallaghan3@gmail.com (D.C.); razia8222@yahoo.com (R.S.); 2 Department of Pharmacology and Physiology, Georgetown University, 3900 Reservoir Road, NW, Washington, DC 20007, USA; 3 Department of Pharmacology and Physiology, School of Medicine, George Washington University, Ross Hall 2300 Eye Street, NW, Washington, DC 20037, USA

**Keywords:** calcium phosphate, kidney, calcification, physiologic, calculi, ossification, ectopic

## Abstract

Calcium phosphate (CaP) crystals are formed in pathological calcification as well as during stone formation. Although there are several theories as to how these crystals can develop through the combined interactions of biochemical and biophysical factors, the exact mechanism of such mineralization is largely unknown. Based on the published scientific literature, we found that common factors can link the initial stages of stone formation and calcification in anatomically distal tissues and organs. For example, changes to the spatiotemporal conditions of the fluid flow in tubular structures may provide initial condition(s) for CaP crystal generation needed for stone formation. Additionally, recent evidence has provided a meaningful association between the active participation of proteins and transcription factors found in the bone forming (ossification) mechanism that are also involved in the early stages of kidney stone formation and arterial calcification. Our review will focus on three topics of discussion (physiological influences—calcium and phosphate concentration—and similarities to ossification, or bone formation) that may elucidate some commonality in the mechanisms of stone formation and calcification, and pave the way towards opening new avenues for further research.

## 1. Introduction

Calcium ions (Ca^2+^) are an abundant mineral inside the human body, stored as calcium phosphate (CaP) in the skeleton, where it serves to maintain healthy bones and teeth. Little amounts of calcium are also dissolved as ionized Ca^2+^ in the intra- and extracellular fluid of every cell, which is essential for the normal functioning of the heart, muscles, blood vessels, and nerves [1]. In order to achieve these functions, our body constantly regulates those Ca^2+^ levels [1]. Ca^2+^ can also act as signaling molecule by acting both as first [2] and second [3] messenger to drive many of the intracellular signal transduction pathways. However, Ca^2+^ can build up as CaP, either in the form of brushite, hydroxyapatite, or other apatites, in places where it does not belong, *i.e.*, in the soft tissue, called ectopic calcification, which can cause several life-threatening diseases including nephrolithiasis and atherosclerosis [4,5]. Interestingly, some of these CaP deposits originate in tubular microenvironments within a hollow tissue structure (such as in kidney tubule and in salivary duct), and thus there may be some common factors that can influence the process of CaP crystal deposits, leading to stone formation [5,6,7]. Moreover, CaP crystals, which can form within blood vessels and the kidney tubular system, involve bone-related factors such as bone morphogenetic proteins (BMPs) and osteopontin (OPN) in the crystal matrices through an upregulation of transcription factors runt-related transcription factor 2 (Runx2) and msh homeobox 2 (Msx-2) [8].

The core idea of the mechanism of calcium biomineralization remains centered on pH, and physiological phosphate and calcium concentrations. However, to date, the stimuli for CaP deposition and the precise mechanism by which these crystals grow into stones are still poorly understood. Some have suggested that preformed microcrystals attach to the surface of, for example, kidney tubular cells, which then leads to further deposition of crystals and the subsequent formation of full-sized stones [9,10]. Other researchers have emphasized the so-called free solution crystallization mechanism, whereby a continuous crystallization process, although periodically inhibited by organic molecules, eventually leads to the materialization of a nucleus from which stones form [9,10]. Moreover, different factors affect CaP deposition and stone formation, such as the supersaturation of unbound calcium and phosphate ions or the absence of inhibitors that prevent crystal formation, which pose a challenge to predict the biomineralization process [11].

Accordingly, our review examines the most recent research on CaP crystal deposition, a contributing factor in vascular calcification [12] and in the process of salivary and calcium kidney stone formation [13,14], and their potential commonalities, specifically: (1) physiological influences; (2) calcium and phosphate concentration; and (3) similarities to ossification or bone formation. For example, some studies have found that CaP nanocrystal deposition may induce the osteogenic transformation of vascular smooth muscle cells by upregulating *BMP2* and *OPN* expression, genes associated with vascular calcification [12,15,16], CaP crystal exposure to renal epithelial cells [8] and bone ossification [17]. Additionally, vascular calcification, which is now an accepted predictor of adverse cardiovascular events, such as myocardial infarction and stroke, and has been shown to lead to atherosclerosis, and cardiac valve calcification [18], is prevalent among the chronic kidney disease (CKD) patients [19]; interestingly, population studies have shown that symptomatic kidney stone formers are at an increased risk for CKD [20]. One study also demonstrated a possible association between the severity of CKD in young patients and the formation of dental calculus, an additional manifestation of disturbed calcium and phosphate (Ca/P) homeostasis in distant parts of the body [21]. Thus, we believe that a systematic analysis of parallel factors of CaP crystal formation may generate a new understanding of stone development and calcification, and lead to the development of focused research towards new treatment options.

## 2. Physiology Influences the Microenvironment

Physiological factors in the salivary/renal systems and vasculature may affect the microenvironment that could contribute to salivary and calcium kidney stone formation, and calcification. Salivary gland calculi (or sialolithiasis) can lead to the blockage of the gland, and result in persistent swelling and ductal dilation [22], secondary infections, abscess, salivary duct stenosis, and even Kuttner’s tumour, a pseudo-tumour of the submandibular gland accompanied by calcification [23]. Interestingly, a vast majority of salivary calculi occur in the submandibular gland or its duct; the remaining salivary stones develop in the parotid and the sublingual gland [23,24]. The primary reason could be physiological: the submandibular gland has a long duct with two bends, traveling upward and forward, then initiates a radical turn toward the hilus of the gland [25,26]. This slow flow rate against gravity results in saliva stagnation, or “salivary stasis”; the relatively narrower tubule in the Wharton’s duct of the submandibular gland than the Stensen’s duct of the parotid gland also contributes [26]. This higher viscosity in submandibular saliva increases the likelihood of hydroxyapatite calculi and stone formation.

Similarly, differences in renal fluid flow may induce the formation of kidney stones, which have been associated with urinary tract obstruction with acute renal failure, infection, and end-stage renal disease [27]. A recent computer model may provide a clue as to a potential cause. The model incorporated a series of hydrodynamic factors which included the fact that: (1) CaP crystals travelling close to the walls of the descending loop of Henle move at slower velocities than the fluid at the central axis of the tubule; and (2) CaP nucleates as it travels down the renal tubules [28]. This model predicted that, under certain conditions, CaP crystals, which start in the descending loop of Henle, travelling close to the tubular epithelial wall may grow and accumulate [28]. Indeed, nephrocalcinosis, which may be defined as the retention of crystals in the renal tubules, occurs when the tubular epithelial cells become susceptible to crystal attachment [29]. Although under normal conditions, the possibility of crystals growing large enough to be trapped is very small due to the short transit time, hydrodynamic factors (for example, fluid drag close to tubule walls and the gravitational effect on particles travelling upward in the tubule) may lead to a delay of crystal passage long enough to grow and become trapped in the collecting duct, and lead to the formation of Randall’s Plugs in the Ducts of Bellini [28], in which brushite and other CaP stones in primary hyperparathyroidism have been found attached [8].

Thus, the conventional approaches to studying CaP stone formation using two-dimensional cell cultures fail to consider the microenvironmental mechanisms of stone formation. Because such stone formation occurs in a dynamic microenvironment, which requires luminal properties with dynamic pressure-flow kinetics over the microenvironment, the absence of all these critical factors severely limits the capability of understanding the true mechanism underlying the biological process of stone formation. Moreover, stone formation is a multifactorial mechanism [30,31], therefore an *in situ* analysis with all of these factors, which are intertwined with each other, along with the overwhelming complexity of cellular behaviors, regulatory pathways, and differences in physiological interactions poses a grand challenge in understanding such biomineralization process. Since CaP crystal formation is also a multifactorial process, examining one factor alone with several unknowns would not be helpful in delineating the crystallization process. Therefore, a step-by-step analysis of the process in a defined system is vital to the understanding of CaP crystal formation and aggregation, which can lead to stone formation and calcification. A microfluidic (MF) approach, one that simulates an *in* *vivo-*like tubular system and achieves previously unattainable precision in analysis and control over the spatiotemporal biological conditions, have been described [32,33]. Among these establishing polarized epithelia with perfectly cylindrical scaffolds in MF offers an effective means for the recreation of 3D non-leaky polarized monolayer of renal/salivary tubular structures with *in vivo-*like permeability and barrier functions have recently been demonstrated, which can allow a systematic evaluation of the dynamic microenvironmental cues (e.g., cellular regulations, fluidic hydrodynamics) that are critical for CaP stone formation [33].

Additionally the physiological features of the vasculature play a role in stone formation and calcification. Interstitial CaP deposits (Randall’s Plaque) were found to have been attached to a majority of calcium oxalate stones in human idiopathic calcium oxalate stone formers, who represent about 75% of all stone formers [34]. One theory regarding the development of Randall’s plaque centers on the vasculature. Stoller and others have proposed that the flow of blood from an intact endothelium in the descending vasa recta to the fenestrated ascending vasa recta decreases both vascular resistance and flow velocity in the ascending vasculature, predisposing the renal papilla to injury [35]. Each descending vasa recta “approaches the papillary tip and makes a hairpin turn,” bifurcating into four ascending vasa recta, changing the blood flow from laminar to turbulent, and increasing the likelihood of injury at the renal papilla’s vascular environment [35]. This vascular injury may subsequently augment Randall’s plaque precipitation [36]. It has also been proposed that the repair of renal papillary vasculature leads to calcification near the vessel walls and may ultimately form a calculus into the papilla [37]. Interestingly, it has been suggested that the repair of this injury to the renal papilla is similar to the atherosclerotic-like reaction in damaged vessel walls, which has shown to lead to the calcification of the vessel wall [35,37]. Additionally, some have proposed that atherosclerotic plaque, in which calcium deposits in the form of hydroxyapatite were found within [38,39] and the site where coronary arterial calcification occurs [39,40], form at the arteries that are most disposed to injury, *i.e.*, sites where arteries bifurcate, similar to vasa recta bifurcation in the kidneys.

## 3. Calcium and Phosphate Concentrations

### 3.1. Salivary 

Salivary gland stones have been found to be composed of different CaP. Hydroxyapatite is traditionally thought to make up a large part of salivary gland stones. Recent studies also suggest that salivary calculi are made up of other apatites, including amorphous carbonated CaP and carbonated apatite and whitlockite. In addition to the CaP, these calculi are also composed of an organic matrix, including fibrous proteins that can be found in greater than 60% of calculi and in fact can make up 60% to 95% of a given stone, which accumulate around the CaP serving as a nucleus [41,42,43].

The build-up of CaP crystals is the nidus for the development of salivary gland stones. While the precise mechanism may not be understood, there are certain etiological factors that are known to contribute to the formation of salivary calculi, such as the deposition of calcium salts around a core comprised of shed epithelial cells [23]. This makes sense since higher concentrations of saliva calcium have been shown in patients who develop hydroxyapatite calculi [41]. Moreover, experimental and clinical studies strongly suggest a link between high calcium concentration ([Ca^2+^]) in saliva and sialolithiasis. Sialolithiasis starts with the formation of calculi; patients with calculi and sialolithiasis are shown to have higher [Ca^2+^] in their final saliva [44]. Additionally, total saliva [Ca^2+^] levels have been shown to be higher (~20 mg/L) in patients with calculi [41]. Evidence also indicates that calcium is the major component of salivary stones [43,45,46]. This, therefore, suggests that [Ca^2+^] in ductal saliva is critical in the development of salivary gland stones [47].

**Figure 1:**
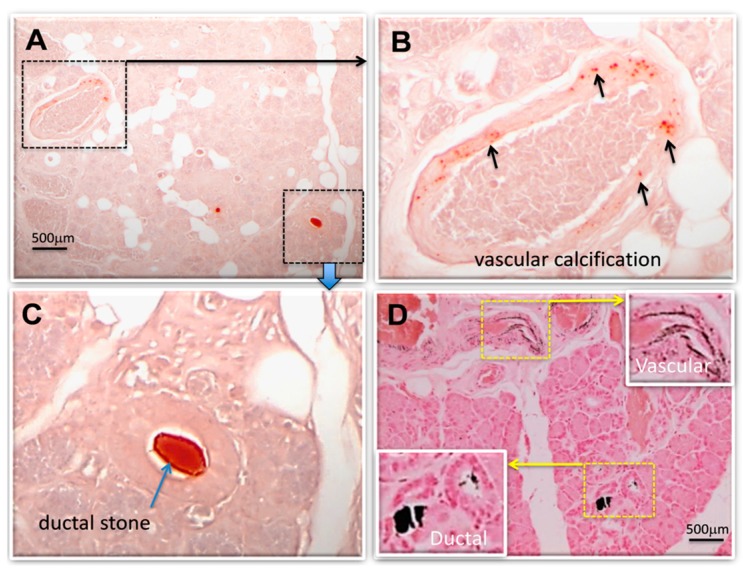
Vascular and ductal calcifications were found in salivary gland from stone patient. Human submandibular gland (SMG) sections from stone patient were used to detect calcified material. Calcified blood vessel and intra-ductal stones were stained with the: (**A**) alizarin red method [48] for examining the calcified deposits; enlarged in (**B**) showing vascular calcification; and (**C**) showing intra-ductal stone (pictures are representative of *n* = 6); no such staining was found in control SMG (*n* = 4). Von Kossa [49] stained SMG (**D**) confirms the presence of calcification, both vascular and stone, in same SMG tissue. Specimens are formalin fixed paraffin embedded (FFPE) de-identified tissue section (biopsy sample, not individually identifiable to any person) from tissue bank through an exempt Institutional Review Board (IRB) protocol.

Interestingly, we have identified vascular calcifications in human submandibular (SMG) salivary gland section from a stone patient (Figure 1). Indeed, both vascular calcification and intra-ductal stones are detected in the salivary gland tissue sections (Figure 1), indicating an organ specific deregulation of both Ca^2+^ and PO_4_^3−^ in salivary stone conditions. CaP, in the form of hydroxyapatite, is the major inorganic component in salivary stones [44] and CaP deposition is critical to vascular calcification [12]; this indicates that the overall levels of Ca^2+^ and PO_4_^3−^ in serum and the saliva could play in the calcification/stone forming mechanism. In support of this view, researchers already found that saliva Ca^2+^ of sialolithiasis patients is higher [44], which favors the crystallization mechanisms. However, what triggers the vascular calcification in such case is unknown. Additional mechanistic research is needed to provide further understanding of the molecular mechanism of calcium stone formation and calcification.

### 3.2. Kidneys

Research has shown that increases in calcium and phosphate concentration may contribute to the formation of CaP crystals that eventually lead to stone development. A recent computer model has indicated that a possible cause of spontaneous tubular CaP crystal formation, specifically at the descending loop of Henle, may either be due to an increase in plasma phosphate, or a decrease in phosphate fractional reabsorption in the proximal tubule [28]. Additionally, in a recent study that found a greater incidence of tubular plugging, in which CaP stone formers (brushite and apatite) exhibited severe plugging, CaP supersaturation was one factor that was associated to these plug formations [50]. Indeed, hypercalciuria is considered the most common abnormality in calcium nephrolithiasis patients [51] and has been frequently associated with Randall’s plaque formation [52].

In animal models, sodium–hydrogen exchanger regulatory factor-1 (NHERF-1) null mice, which are hypercalciuric and hyperphosphaturic, produced interstitial CaP deposits in the form of apatite in the renal papilla [8,51]. Although the site of these depositions in NHERF-1 null mice were similar to those found in human idiopathic calcium oxalate stone formers [53], no direct evidence of NHERF-1 mutation has shown to cause kidney stones in humans. However, a clinical study observed that humans with a loss-of-function NHERF-1 mutation exhibited hypophosphatemia, indicating decreased capacity for kidney tubule reabsorption of phosphate, which increases the risk of renal calcium lithiasis [54]. Hypophosphatemia can then lead to an increase in intestinal phosphate and calcium absorption through increased 1,25-dihydroxyvitamin D [1,25(OH)_2_ D] production [55], leading to hypercalcemia and hypercalciuria, a risk factor for calcium renal stone formation [56].

Another animal model has shown that kidney type-IIa sodium-phosphate (Na-Pi) co-transporter (Npt2a) null mice exhibited a production of CaP crystal deposits in the kidney tubules and interstitium [8,57]. It is possible that CaP crystals may have originated in the tubular lumen but was eventually displaced to the interstitium, as has been shown in certain Npt2a null mice models, and suggested by clinical and other animal studies [58]. However, no evidence has been found of this to occur in human cases; indeed, the pathogenesis of interstitial CaP deposits of Npt2a null mice are seemingly different from Randall’s Plaque in humans [58]. Interestingly, though, disrupted Npt2a null mice exhibited an adaptive increase in 1,25(OH)_2_ D serum concentration, and associated hypercalciuria and renal calcification [59]. The same may also occur in humans with Npt2a mutations. One study in humans showed that Npt2a mutations led to the impairment of phosphate reabsorption, causing hypophosphatemia [60]. This may eventually result in low phosphate concentration in the serum and the expected increase in 1,25(OH)_2_ D serum concentration [61,62], hypercalciuria [60,63,64], and the formation of different CaP complexes [55]. Indeed, the combination of hypercalciuria, whether from increased intestinal calcium absorption [55,63] or idiopathic hypercalciuria [64], and hyperphosphaturia may lead to the formation of calcium phosphate complexes, and subsequently, nephrolithiasis.

### 3.3. Vascular

Elevated levels of calcium and phosphate in the serum can increase the deposition of CaP in the vasculature. For example, calcification found in the vascular system has been linked to hyperphosphatemia and increased calcium-phosphate products [65,66,67]. Also, *in vitro* studies have found that in response to elevated extracellular calcium ([Ca^2+^]_o_), the expression of the calcium sensing receptor (CSR), a G-protein coupled receptor that evokes signaling pathways according to changes in [Ca^2+^]_o_ concentration, had been downregulated in vascular smooth muscle cells induced to calcify, suggesting that ablation of CSR function was associated with increased calcification in vascular smooth muscle cells [67,68]. It is believed that, without the function of CSR, vascular smooth muscle cells are unable to respond to the increase in calcium, and produce the necessary inhibitory proteins to prevent calcification [68]. Moreover, one study has shown that elevated calcium concentrations, even under normal phosphate levels, induced the mineralization of human smooth muscle cells, and then was accelerated by increased phosphate levels [69]. Increases in calcium and phosphate also induced calcification in human vascular smooth muscle cells [70]. In both cases, the presence of elevated calcium and phosphate exacerbated calcification [71].

Intriguingly, in both animal and clinical studies, it has been shown that elevated phosphate levels indeed played a role in osteogenic and chondrogenic differentiation, which eventually leads to the formation of a complex and highly structured extracellular matrix [68] found in calcified vasculature. It has been shown that inorganic phosphate uptake by vascular smooth muscle cells induces transcription factors related to bone ossification, *i.e.*, Core-binding factor α(1), Cbfα1, also known as Runt-related transcription factor 2 (Runx2) [72,73]. Moreover, studies have shown that high phosphate cultured with vascular smooth muscle cells *in vitro* can calcify, and contain phenotypic transitions to several types of cells association with bone formation, such as osteoblasts and chondrocytes [18,73,74,75,76,77,78]. Thus, increasing evidence such as these indicates that arterial calcification is also an active process that shares many similarities with bone formation [16].

Interestingly, hypercalcemia and hyperphosphatemia can further exacerbate vascular calcification initiated by lipids. Indeed, Demer noted that lipids have an in important role in both bone calcification and calcification in atherosclerosis [79]. For example, researchers noticed through confocal microscopy that there was cholesterol within the calcified granules of atherosclerotic plaque, which suggest that lipids may nucleate calcium crystals [79,80]. Similarly, within the matrix vesicles, the nidus for crystallization is phospholipids, which are purposed to initiate and regulate hydroxyapatite formation [81].

## 4. Vascular Calcification and Stone Formation Similarities to Bone Mineralization

### 4.1. Overview of Bone Formation

There are two types of ossification: intramembranous and endochondral. Intramembranous ossification involves the formation of bone directly from mesenchymal tissue, primarily forming bones of the skull, clavicle, and mandible [17], and has been noted to be essential in the healing of compound fractures [81]. In endochondral ossification, mesenchymal cells are converted to bone through a cartilage intermediate [17], and are involved in the formation of long bones such as the femur, tibia, humerus, and radius [81].

Intramembranous ossification is initiated by mesenchymal stem cells and involves BMPs and transcription factor Cbfα1/Runx2. BMPs, such as BMP2, BMP4, and BMP7, activate the *Cbfα1* gene in mesenchymal cells to transform them into osteoblasts [17]. A study has shown that Runx2/Cbfα1 not only regulates osteoblast differentiation by activating major bone matrix genes [82] but also is necessary to induce osteoblastic differentiation in undifferentiated mesenchymal cells into osteoblasts [83], where Cbfα1 expression requires the activation by BMP2 [84]. BMP2 has shown to induce ectopic bone formation *in vivo* [85], regulate osteogenic differentiation *in vitro* [85], and mediate bone fracture repair [86]. Conversely, Runx2 null mice did not demonstrate intramembranous or endochondral ossification [87,88], and BMP blocking antibodies inhibited Runx2 osteoblastic differentiation [89]. Thus, Runx2/Cbfα1 [90] and BMP2 [86] have been shown to be integral in osteoblastic bone formation. In fact, *Bmp2*^+/−^, *Bmp6*^−/−^ mice displayed a reduction in bone formation in primary spongiosa, which Kugimiya and others believed was probably due to reduced bone formation [85]. Furthermore, BMP ligand deletion has shown to weaken chondrogenic or osteogenic differentiation [91]. Interestingly, one study found that BMP2 stimulates vascular smooth muscle cell differentiation into an osteoblastic phenotype; moreover, the BMP2 effect was reversed by β-catenin knockout, which implies that a second major pathway in osteoblastogenesis, the Wnt/β-catenin signaling pathway, is involved in BMP2 induced vascular smooth muscle cell differentiation into an osteogenic phenotype and subsequent calcification [92].

In endochondral ossification, rather than differentiating directly into osteoblastic phenotype, mesenchymal cells form cartilage tissue as an intermediate [17]. Transcription factors, Pax1 and Scleraxis, activate cartilage-specific genes [93,94] to commit the mesenchymal cells into differentiating into chondrocytes [17]. In fact, *Sox9* gene has been expressed in these precartilaginous condensations, and is quintessential in the regulation of chondrocyte development [95,96,97]. Studies have shown that a defect in the *Sox9* gene results in bone deformities in the body [17]. Interestingly, the repression of *Sox9* suppresses reprogramming of descending aorta vascular smooth muscle cells into chondrogenic phenotype, and the potential calcification of the vascular wall [98]. Continuing in the endochondral ossification process, chondrocytes then rapidly proliferate and enlarge their volume by secreting an extracellular matrix; the enlarged chondrocytes add collagen X and fibronectin to ensure it is mineralized by calcium carbonate [17]. All of the cartilage is eventually replaced by bone.

### 4.2. Kidney 

The previous discussion of the kidneys highlighted that the physiological features of the vasculature, and high calcium and phosphate concentrations, may lead to the deposits of CaP in the interstitium. Interstitial CaP deposits, which are found in calcium oxalate stones in idiopathic calcium oxalate stone formers [4], may subsequently lead to the development of Randall’s plaque, initially formed when a CaP spherule is deposited in the loops of Henle, collecting ducts, or vasa recta [99]. However, not all of Randall’s plaque can lead to stone formation [99]. Interestingly, it has even been proposed that Randall’s plaque may be an example of nephrocalcinosis [100]. Nevertheless, the formation of stones requires the complex integration of numerous factors, for example, the formation, retention, and accumulation of crystals; mineral (*i.e.*, calcium and phosphate) supersaturation; urinary pH; and a disruption, reduction, or abnormalities in crystallization inhibitors [99]. Interestingly, in a recent retrospective study, one specific type of papillary stone (calcium oxalate monohydrate, COM) was found to have developed from crystals or organic matter that was attached to the renal papilla [101]. Indeed, it is believed that renal subepithelial papillary hydroxyapatite calcification is involved in COM stone formation [102], with recent evidence showing that hydroxyapatite crystals in the papillary, which can form into Randall’s Plaque, can certainly become the nidus of a COM papillary stone [103].

Thus, the formation of Randall’s plaque is particularly interesting, made more so because the involvement of membranous vesicles and collagen is similar to the mechanisms of other forms of ectopic calcification [104]. This may help to explain the association between kidney stones and subclinical atherosclerosis in young adults and the observation that these diseases share many of the same factors [105]. A new retrospective, matched case-control study by Shavit and others has found that patients who recurrently formed calcium kidney stones had higher incidences of aortic calcification, which suggests that stone formation and vascular calcification are linked [106]. Moreover, these [105] and other authors [107,108,109,110,111] found that patients who formed calcium kidney stones exhibited lower bone mineral density, suggesting that bone demineralization, which often accompanies hypercalcemia [112], may be linked to calcium stone formation. In fact, a previous study found increased occurrences of osteopenia and osteoporosis in patients with recurrent calcium nephrolithiasis and hypercalciuria [113]. Interestingly, as Gambaro *et al.* has pointed out, lower bone density has also been linked to abnormal arterial stiffness, partially due to calcification, which is a strong indicator of cardiovascular mortality [114]. These studies seemingly provide a link between bone, kidney calcium stone formation, and arterial calcification.

Moreover, a number of studies have found bone-related proteins, and transcription factors related to bone ossification, in kidney calcium stone formation. For example, evidence has shown that kidney epithelial cells can de-differentiate into an osteoblastic phenotype, increasing production of bone-specific proteins such as OSN, OPN and bone sialoprotein (BSP), supporting the nucleation of CaP crystals [8,115]. Moreover, increased levels of BMP2 and transcription factors has been linked to osteoblastic differentiation and bone formation, such as Runx2, to kidney CaP deposits in idiopathic hypercalcuria, where 1,25(OH)_2_ D_3_/vitamin D receptor (VDR) plays an important role in its regulation [116]. These connections provide a potentially novel relationship between bone formation, and crystal deposition and formation in the kidneys. Furthermore, new research has found that Ca^2+^ augments the response of transforming growth factor β1 (TGF-β1) to produce BMP2 and OPN in human kidney proximal tubule epithelial cell line, HK2, and in primary renal epithelial cells (PRECs) of nephrolithiasis patients with idiopathic hypercalciuria [117]. It has even been suggested that serum BMP2 levels, in combination with cystatin C, may be a potential biochemical marker for stone formation [118]. These findings suggest the important role of increased Ca^2+^ in the pathogenesis of stone formation and accentuate the potential relationship between kidney stone formation and bone mineralization/de-mineralization.

### 4.3. Vascular

**Figure 2:**
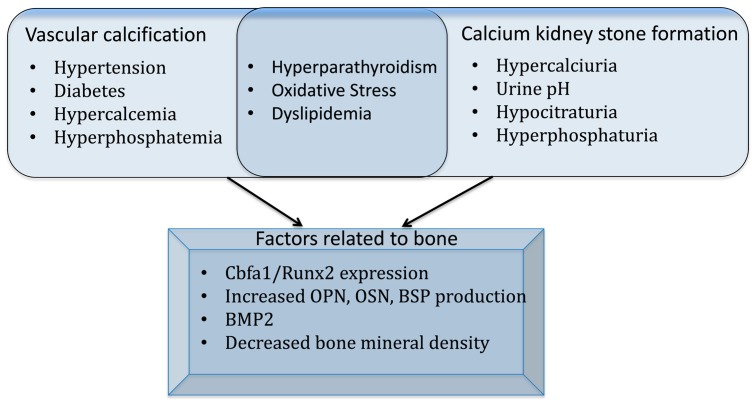
Identified instigators of vascular calcification and stone formation, highlighting common factors, and several commonalities related to bone mineralization/de-mineralization. Risk factors for vascular calcification include both the “traditional” (such as hypertension and diabetes) and the “non-traditional” (*i.e.*, abnormal mineral metabolism such as hypercalcemia and hyperphosphatemia) [123]. A majority of calcium kidney stones are predominantly calcium oxalate, with CaP admixed in small amounts [4]. Thus, factors associated with the predominant form of calcium stone formation include: hypercalciuria; urine pH, high (pH > 6.3) for CaP stone formation, and for CaP deposits that may be the initial nidus for calcium oxalate stones, and low for calcium oxalate stone formation; and hypocitraturia, which increases the chance for calcium to bind with oxalate or phosphate [4]. Hyperphosphaturia may increase the risk of calcium kidney stone formation by urinary calcium excretion and urinary saturation [56]. Common risk factors for both vascular calcification and calcium kidney stone formation include hyperparathyroidism [4,123], oxidative stress [99,123], and dyslipidemia [37,123]. Interestingly, studies have found bone related proteins and other bone related factors in vascular calcification, calcium deposits and calcium kidney stone formation as discussed in the review.

Although vascular calcification was believed to be a passive process, it is now accepted that arterial calcification are active and highly regulated forms of calcification [119], similar to bone formation [16] (Figure 2). Indeed, magnetic resonance spectroscopy studies indicate that the hydroxyapatite structure in calcified vessels were similar to the hydroxyapatite in bone [120]. It has been proposed that vascular calcification is initiated when the cells de-differentiate into osteoblast-like phenotypes [121]. Indeed, cells (*i.e.*, pericytes in microvessels; pericyte-like, calcifying vascular cells in the aortic intima; smooth muscle cells in media; and myofibroblasts in the adventitia) have been isolated from vascular tissue and have been found to undergo osteoblastic de-differentiation [121]. These cells may originate from similar mesenchymal stem cells as osteoblasts, which themselves are induced with the transcription factor Runx2 or Msx-2 [122]. Although this is believed to be a common mechanism in both types of calcification, the factors that initiate this de-differentiation may differ [122].

Specific factors that lead to intimal calcification in atherosclerosis include inflammatory factors produced within the actual atherosclerotic plaque, including modified lipoproteins and cytokines [121,124]. Notably, patients with homozygous familial hypercholesterolemia, due to mutations in the LDL receptor (LDL-R), develop aortic calcification independent of the levels of total cholesterol [121]. This has led to the hypothesis that LDL-R modulates the osteogenic signaling pathways, such that a mutation at the LDL-R gene locus leads to the dysregulation of the calcification signaling pathways in the osteoblast-like cells and the activation of Msx-2 and Runx2 [121]. Additionally, hydroxyapatite crystals within atherosclerotic plaques themselves may promote further calcification by inducing monocytes that ingest them to produce inflammatory cytokines [119]. Ultimately, in arterial intimal calcification, the Runx2/Cbfa-1 and Sox9 pathways predominate, although Msx-2 expression has been found in arterial specimens that include diabetic patients [125].

Whereas the Runx2/Cbfa-1 pathway predominates in intimal calcification, the early stages of medial calcification have been that the Msx-2 and Wnt pathways predominate [121]. Hyperglycemia also promotes calcification via enhanced alkaline phosphatase expression via the Runx2/Cbfa-1 pathway [121]. Arterial medial calcification is also induced in renal insufficiency by high serum levels of phosphate and calcium, which promote the Runx2/Cbfa-1 pathways. Furthermore, inorganic phosphate directly promotes osteoblastic phenotype transformation in vascular smooth muscle cells (VSMCs) by inducing a sodium-dependent phosphate transporter [12,121]. After the phenotype change, the osteoblastic cells also induce calcification via expression of other bone-regulating proteins include OPN, osteocalcin, OSN and type II collagen [126]. Additionally, these osteoblastic cells secrete matrix proteins, which serve as the foundation upon which calcification occurs both through the secretion of matrix vesicles containing preformed apatite [127,128] or through further apoptosis [124,127,129,130]. Apoptotic cell death increases membranous debris rich in phospholipids that can be the conduit to nucleate apatite, particularly in individuals with atherosclerosis and other diseases with widespread necrosis and apoptosis.

Once the matrix vesicles are secreted, increased levels of phosphorus and calcium can increase their mineralizing potential, which may explain why calcification is more prominent in individuals with chronic kidney disease and thus causes disturbances in mineral (*i.e.*, calcium and phosphate) metabolism [122]. Normally, mineralization through said matrix vesicles is prevented through mineralization inhibitors (such as matrix Gla protein and fetuin-A), but in cases where there are diminished or ineffective mineralization inhibitors, the matrix vesicles can then serve as niduses for further calcification [127,128]. 

Interestingly, aortic valve (valvular) calcification has been suggested to possess similar characteristics of arterial (vascular) calcification [126]. Indeed, similar to VSMC, valvular interstitial myofibroblast-like cells showed expression of OPN, osteocalcin, and Runx2, which suggests an active mineralization process prior to the development of end-stage calcification (*i.e.*, macrocalcification) [126] involving valvular endothelial cells, valve interstitial cells, inflammatory cells, and the extracellular matrix [131]. Some studies have associated valvular calcification with a faster progression of aortic stenosis which clinically manifests into exertional angina, syncope, and heart failure [131], and worse morbidity and mortality [132]. Additionally, vascular calcification is common in end-stage renal disease (ESRD) patients [133], due to their elevated risk of mineral metabolism disturbances [134]. Interestingly, it has been proposed that valvular calcification may be marker of arterial calcification in ESRD patients [134].

### 4.4. Breast

Ectopic calcification in the breast is a telltale sign of breast cancer, and is one of the distinguishing features examined for on diagnostic mammography. It has been reported that microcalcifications can be found in up to 88% of non-palpable tumors [135], and up to 93% of ductal carcinoma *in situ* (DCIS) cases are presented alongside microcalcification [136]. Thus, these microcalcifications can be used to detect breast cancer in its early stages. However, studies have shown that microcalcification is being associated with worse prognoses; indeed breast cancer presenting with microcalcification are more associated with lymph node invasion and HER-2 positivity [136]. Similar to kidney calcification, breast microcalcification comes in two forms, known as Type I and II crystals. Type I crystals are composed of calcium oxalate and are more frequently associated with benign ductal cysts [137]. Type II crystals are composed of hydroxyapatite and are associated with both benign and breast cancer lesions [138]. Furthermore, as the carbonate content of the hydroxyapatite crystals decreases, the grade of the lesion increased [139]. Indeed, it is hypothesized that the hydroxyapatite itself increased the grade of the cancer because hydroxyapatite has been found to increase the mitogenesis of the cancer cells [140].

**Figure 3:**
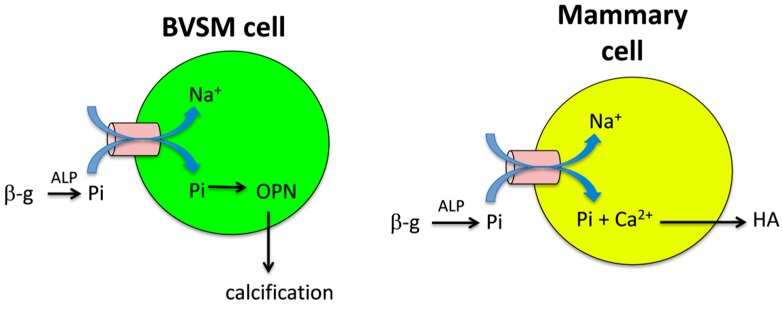
In bovine vascular smooth muscle (BVSM) cell and mammary cells, studies have suggested that Na-Pi co-transporters can induce calcification and hydroxyapatite (HA) crystal production, respectively. In BVSM and mammary cells, β-glycerophosphate (β-g) via alkaline phosphatase (ALP) hydrolyzes pyrophosphate (Ppi) into Pi, where Pi is transported into the cell via Na-Pi co-transporters [136,144]. In BVSM cells, the increase in Pi induces osteopontin (OPN) expression and calcification [144]. In mammary cells, increased in intracellular Pi combines with Ca^2+^ to product HA crystals; however, further research is needed to determine the mechanism in which HA crystals are transported into the extracellular matrix [136].

Until recently, very little information was known about the mechanism of hydroxyapatite formation in breast cancer, largely because of a lack of a reproducible, high-penetrance animal model available to study this process [141]. It was not even known whether the calcification was a result of an active or passive process. However Cox and others developed a reproducible *in vitro* model using the metastatic mammary adenocarcinoma 4T1 cell line that ultimately allowed them to propose a mechanism for hydroxyapatite calcification [136]. They hypothesized that alkaline phosphatase (ALP) not only hydrolyses β-glycerophosphate to glycerol and phosphate (Pi) [136], which is similar to the initial osteoblastic differentiation process in ossification [142], but ALP also dephosphorylates OPN and hydrolyzes pyrophosphate (PPi) to Pi on the surface of mammary cells [143]. The produced Pi is then believed to be transported into the 4T1 cells by type II Na-Pi co-transporters, which have been found to be expressed in higher levels in cancer tissue as opposed to normal tissue [136]. When inside the cell, the Pi combines with calcium to ultimately produce hydroxyapatite crystals [136]. However, further research will be necessary to ascertain how these crystals eventually are transported out of the cell to the extracellular matrix. Mineralization was prevented when these Na-Pi co-transporters were inhibited [143], which suggests that this calcification is an active process in the breast cancer cells. Interestingly, β-glycerophosphate was found to cause elevated OPN expression and calcification in bovine vascular smooth muscle cells, which were prevented by the inhibition of type III Na-Pi co-transporters [144]. This suggests that Na-Pi co-transporters play a role in the calcification and CaP (in the form of hydroxyapatite) deposition process in vascular and mammary cells, respectively (Figure 3).

Moreover, Cox and others later determined that, during this process of mineralization, there is no change in the amount of Runx2 mRNA [143], which also a role in bone ossification and is seen in vascular calcification. With that said, other transcription factors do exist and their involvement cannot be ruled out at this time. A separate study also suggests that BMP2 may be involved after determining that BMP2 can induce microcalcification in adenocarcinoma cells while at the same time sparing normal tissues, possible through the formation of osteoblast-like cells being derived from pericytes [141]. Additionally, studies have determined that both *in situ* and invasive breast carcinomas had increased levels of OSN, OPN, and BSP, which were also associated with higher levels of microcalcification [145,146]. Increased expression of OPN mRNA further suggests that this process is similar to physiological osteoblasts [143]. Indeed, one recent study suggested breast epithelial cells may acquire mesenchymal characteristics, where osteoblast-like cells secrete microcalcifications in the form of hydroxyapatite into the extracellular space [147].

## 5. Conclusions

CaP crystal deposition has been identified to be the source of calcification and stone formation, both of which may lead to potential adverse conditions. Thus, a comparative review of the mechanism in which CaP crystal aggregates may provide important insights. For one, because such stone formation occurs due to changes within similar hollow tubular structures, it intimates an avenue of commonality among other sites of crystal formation [5,6,7,9,35]. Thus, further studies are needed, whether *in vivo*, or *ex vivo* utilizing microfluidics, to determine whether changes in the spatiotemporal compositions of the biological fluids may impact other sites of hollow tubular structures, such as the breast and pancreatic ducts.

Secondly, as suggested in this review, high [Ca^2+^] levels, for example, in the saliva and in kidney luminal fluid, may potentially develop into salivary calculi and CaP crystals [8,57,59], respectively. Studies have shown an association between increased [Ca^2+^]_o_ with increased expression of bone-specific proteins involving Ca^2+^ signaling. Elevated [Ca^2+^]_o_ levels have been shown to stimulate osteoblastic differentiation, but one study performed by Koori and others has shown that increased [Ca^2+^]_o_ levels also increase bone-related gene expression and calcification [148]. Interestingly, inhibiting the CSR induced greater bone-related gene expression and calcification; further antagonizing CSR had shown to enhance the role of calcium in osteogenic differentiation [148]. Additionally, one study has indicated that BMP2 may inhibit transient receptor potential canonical (TRPC) 1, TRPC4, and TRPC6 expression, thereby regulating Ca^2+^ signaling and decreasing intracellular Ca^2+^ levels in pulmonary arterial smooth muscle cells [149]. Interestingly, it has also been proposed that the dysregulation of Ca^2+^ homeostasis and the disruption of the coordinated action of calcium pumps, channels, sensors and buffers in breast epithelial cells may be involved in the formation of the mesenchymal cell type that has been associated with breast cancer metastasis [150]. Thus, more studies are needed to determine the molecular mechanism that leads to calcification and stone formation due to high [Ca^2+^] levels.

Third, as Khan and others have suggested, studies have shown an increasing role of bone-specific proteins in calculi formation in the kidneys and cardiovascular system [8]. Studies have also shown that OPN can induce hydroxyapatite development [151]. A high phosphate diet has been found to induce OSN expression in the kidney tubules, suggesting that increased OSN expression is involved in the kidney formation of calcium deposits initiated by a high phosphate diet [152]. These same bone-specific proteins are linked to ectopic microcalcification in adenocarcinoma cells as well, suggesting similar mechanisms of crystallization formation in different parts of the body. Thus, there could be a potential link between microcalcification in adenocarcinoma cells, initiated by bone-specific proteins, and carcinoma. Accordingly, further research is required.

Additionally, research has indicated that BMPs, and transcription factors Runx2/Cbfα1, are involved in the deposition of CaP crystals in the kidney, which can eventually lead to stone formation, and in the initial formative stages of vascular calcification. This interesting link to ossification begs to ask whether other CaP deposits in the ducts of the salivary gland, breasts, and pancreas (all which lead to microcalcification and stone formation) are similar to the ossification mechanism. Further research may provide subtle clues that could enhance our understanding of the early stages of stone formation and calcification.
